# Improved Resistance Prediction in *Mycobacterium tuberculosis* by Better Handling of Insertions and Deletions, Premature Stop Codons, and Filtering of Non-informative Sites

**DOI:** 10.3389/fmicb.2019.02464

**Published:** 2019-10-31

**Authors:** Camilla Hundahl Johnsen, Philip T. L. C. Clausen, Frank M. Aarestrup, Ole Lund

**Affiliations:** Research Group for Genomic Epidemiology, National Food Institute, Technical University of Denmark, Kongens Lyngby, Denmark

**Keywords:** antimicrobial resistance (AMR), tuberculosis, whole genome sequencing, bioinformatics, resistance prediction

## Abstract

Resistance in *Mycobacterium tuberculosis* is a major obstacle for effective treatment of tuberculosis. Multiple studies have shown promising results for predicting drug resistance in *M. tuberculosis* based on whole genome sequencing (WGS) data, however, these tools are often limited to this single species. We have previously developed a common platform for resistance prediction in multiple species. This platform detects acquired resistance genes (ResFinder) and species-specific chromosomal mutations (PointFinder) associated with resistance, all based on WGS data. In this study, we present a new version of PointFinder together with an updated *M. tuberculosis* database. PointFinder now includes predictions based on insertions and deletions, and it explicitly reports frameshift mutations and premature stop codons. We found that premature stop codons in four resistance-associated genes (katG, ethA, pncA, and gidB) were over-represented in resistant strains, and we saw an increased prediction performance when including premature stop codons in these genes as resistance markers. Different *M. tuberculosis* resistance prediction tools vary in performance mostly due to the mutation library used. We found that a well-established mutation library included non-predictive linage markers, and through forward feature selection we eliminated those from the mutation library. Compared to other similar web-based tools, PointFinder performs equally good. The advantages of PointFinder is that together with ResFinder it serves as a common web-based and downloadable platform for resistance detection in multiple species. It is easy to use for clinicians and already widely used in the research community.

## Introduction

Next generation sequencing is a rapidly evolving field and it is in the process of being adopted as the standard in many clinical and public health settings. Here, it replaces many traditional typing and phenotyping methods such as those for species determination and detection of antimicrobial resistance. Rapid and precise detection of antimicrobial resistance is important for correct treatment, surveillance and control efforts. Antimicrobial resistance occurs either through horizontal gene transfer or by *de novo* chromosomal mutations (Munita and Arias, 2016). In *Mycobacterium tuberculosis* all acquired resistance have been mediated by chromosomal mutations, and horizontal transfer have not been described (Smith et al., 2013). In addition to acquired resistance *M. tuberculosis* have a number of intrinsic resistance mechanisms including modification of drug targets, chemical modification of drugs, enzymatic degradation of drugs, molecular mimicry of drug targets, and drug deportation by efflux pumps (Smith et al., 2013). This is a serious obstacle for effective tuberculosis treatment and prevention of the disease worldwide (World Health Organization [WHO], 2017). Mutations and other genetic changes may lead to enzymatic inactivation of antibiotic molecules, overexpression of novel efflux pumps and porin alterations in the cell wall, trapping of drugs and overexpression of proteins involved in neutralizing the effect of drugs. Due to slow growth rates of *M. tuberculosis*, determining resistance by conventional drug susceptibility testing (DST) is highly time-consuming. Contrarily, next-generation sequencing rapidly yields accurate whole genome sequencing (WGS) data. Using prior knowledge on the genomic changes leading to resistance, WGS data can be used for rapid prediction of antimicrobial resistance (Koser et al., 2014). In fact, studies have already shown promising results for predicting resistance in *M. tuberculosis* based on WGS for first-line anti-tuberculosis drugs (Feuerriegel et al., 2015; The CRyPTIC Consortium and the 100, 000 Genomes Project et al., 2018). However, a challenge for applying this knowledge in a clinical setting is that resistance predictor tools are often limited to a single species. We have previously developed ResFinder (Zankari et al., 2012), an *in silico* method for detection of acquired genes associated with antimicrobial resistance in multiple species based on WGS data. ResFinder was recently expanded with PointFinder (Zankari et al., 2017), a species-specific tool detecting chromosomal mutations associated with drug resistance. PointFinder already includes five species. The rationale of this study is to expand PointFinder also to cover *M. tuberculosis*. In addition to point mutations insertions and deletions may also affect resistance. Especially if the insertion/deletion length is not a multiple of three they will cause the rest of the gene to be read out of frame, which have a high likelihood of introducing a stop codon leading to a truncated gene. We have therefore set out to do a thorough analysis of the correlation of premature stop codons with resistance. In this study we optimized and evaluated the performance of PointFinder’s prediction of resistance in a sixth species, *M. tuberculosis*. *M. tuberculosis* was chosen because of its importance for global health. It is also an organism for which many resistance mutations have been described. Here, we wanted to investigate is some of these are in fact non-informative when it comes to predicting resistance, and study in more detail how the presence of premature stop codons affects resistance. We used a data set of 3,528 *M. tuberculosis* isolates in the optimization which consisted of omitting non-predictive mutations from a well-established mutation library, and including premature stop codons as resistance markers. 2,480 isolates were used to validate the performed optimization.

## Materials and Methods

### PointFinder Database

The PointFinder database contains both a mutation library listing resistance-associated chromosomal mutation and a collection of reference sequences in which these mutations occur. All database files are available at bitbucket.org/genomicepidemiology/pointfinder_db.

The tuberculosis mutation library was obtained from pathogenseq.lshtm.ac.uk, under Tuberculosis and Rapid DR Study and described in Coll et al. (2015). Additional mutations were achieved from a genome wide association (GWA) study performed by the same group in Coll et al. (2018). Mutations, which were observed in the GWA study to be significantly associated with resistance and observed more than 10 times, were also included in the mutation library. All genes, RNA genes and promoter regions of interest for resistance prediction in *M. tuberculosis* are shown in Table 1. Reference sequences for genes and genomic regions listed in Table 1 were obtained from the H37Rv *M. tuberculosis* reference strain, NCBI-reference sequence: NC_000962.3.

**TABLE 1:** Genes and genomic regions of interest for drug resistance in *M. tuberculosis*.

**Drug**	**Genes**	**RNA genes**	**Promoter regions**
Rifampicin	rpoB, rpoC		
Isoniazid	katG, inhA, kasA, ahpC		katG promoter, ahpC promoter, fabGl promoter
Streptomycin	rpsL, **gidB**, embB, embC,	rrs (16SrRNA)	
Ethambutol	embA, embR, **ubiA**		embA promoter
Amikacin		rrs (16SrRNA)	
Capreomycin	tlyA	rrs (16SrRNA)	**idsA2 promoter**
Ethionamide	ethR, ethA, inhA		fabGl promoter, **ethA promoter**
Kanamycin		rrs (16SrRNA)	eis promoter
Pyrazinamide	pncA, panD, rpsA		pncA promoter
Fluoroquinolone	gyrA, gyrB		
Para-aminosalicylic acid	ridD, folC, thyA		**thyX promoter**
Linezolid	rplC	rrl (23S rRNA)	
Bedaquiline	Rv0678		
Clofazimine	Rv0678		
d-Cycloserine	**iniA, alr**		
XDR-TB	**drrA**		**nuoA promoter**

*For each drug the genes and genomic regions of interest for resistance are listed. All gene names refer to a gene sequences in the H37Rv reference genome with the National Center for Biotechnology Information (NCBI) reference sequence: NC 000962.3. The loci in bold are novel resistance-associated genomic regions revealed by a genome wide association study from Coll et al. (2018).*

### PointFinder

PointFinder is both a web service and command line application for predicting resistance associated with chromosomal mutations based on WGS data. The web service is available on cge.cbs.dtu.dk/services/ResFinder/where users can specify to search “Chromosomal mutations” in six different species, including *M. tuberculosis*. The command line version of PointFinder is available on bitbucket.org/genomicepi-demiology/pointfinder.

PointFinder is a Python program that accepts both FastQ and Fasta files for resistance prediction. Initially, the genes of interest for resistance prediction (Table 1) are identified. KMA (Clausen et al., 2018) is used for mapping of raw reads, and BLASTN, RRID:SCR_001598 (Camacho et al., 2009) for aligning assembled genomes, to the genes of interest.

Mutations are detected by comparing the alignments between the reference sequences and the sequences found in the input file. The aligned sequences are compared nucleotide by nucleotide when the alignment represents a promoter region or an RNA gene and codon by codon when it represents a coding gene sequence. Effort has been put into detecting insertions and deletions and reporting any disruption or restoring of the reading frame. If a premature stop codon is detected, this will also be explicitly reported, and no further search for mutations will be performed after the detection of a stop codon. The observed mutations are looked up into the mutation library which holds information about mutations known to be predictive for resistance. If a found mutation exists in the mutation library, the resistance phenotype is returned together with the PubMed ID of the article linking the observed genotype with the predicted resistance phenotype.

### *M. tuberculosis* Data Sets

All data sets used in this study exclusively consisted of paired-end WGS data associated with phenotype data. Phenotype data was given as Resistant or Susceptible based on laboratory determined DST results for multiple anti-tuberculosis drugs.

The first data set, called the ReSeq data set, was obtained from the Relational Sequencing TB Data Platform (Starks et al., 2015). It consisted of WGS data from 3,528 *M. tuberculosis* isolates. The second data set was obtained from the Supplementary Data in Coll et al. (2018) and was used as a validation data set. The validation data set contained 2,480 isolates. The ReSeq and validation data set contained sufficient phenotype data for 10 drugs namely; Rifampicin, Isoniazid, Streptomycin, Ethambutol, Amikacin, Capreomycin, Ethionamide, Kanamycin, Pyrazinamide, and Fluoroquinolones. The number of isolates with determined phenotype varied with each drug. Fluoroquinolones DSTs were determined for the specific compounds namely, Ciprofloxacin, Ofloxacin, Moxifloxacin, and Levofloxacin. However, in the analysis we considered Fluoroquinolones resistance as one, since the mutation library did not distinguish between different compounds. Thus, if an isolate was resistant to any of the Fluoroquinolone compounds it was considered Fluoroquinolones resistant. The data sets can be found in Supplementary Tables S1, S2.

A third data set was used to compare PointFinder to similar resistance predictor tools developed for *M. tuberculosis.* From a scientific report by Schleusener et al. (2017) we obtained 91 isolates that had been used to compare five existing *M. tuberculosis* resistance predictor tools. These 91 isolates were Illumina MiSeq paired end sequenced, and phenotype data existed for five drugs namely, Rifampicin, Isoniazid, Streptomycin, Ethambutol, and Pyrazinamide.

### Measuring Prediction Performance

PointFinder’s detection of resistance-associated mutations was used for binary classification of resistance and susceptibility using the following rules. Isolates were predicted resistant to a drug if one or more mutations predictive of resistance to the drug were found. Isolates were predicted susceptible to a drug if all genes of interest for resistance to the drug were found with an identity above 90% and a sequence coverage above 60%, and no resistance-associated mutations were detected in the genes. We used default options and parameters when running PointFinder. To assess the quality of PointFinder’s binary classification we calculated the Matthew’s Correlation Coefficient (MCC) and the sensitivity and specificity of the prediction.

### Forward Selection of Predictive Mutation

To detect non-predictive mutations, we applied forward feature selection optimized based the MCC over threefold cross-validation. We exclusively examined abundant mutations, defined as mutations found in the ReSeq data set 10 times or more. Mutations found less than 10 times were included in the initial state of the prediction model, whereas the abundant mutations were initially excluded. With each step of the forward selection one abundant mutation was added to the model. The mutation added was the one mutation that benefited the prediction the most based on the MCC. Mutations were added to the model one by one until adding any remaining mutations would decrease the quality of prediction. Examined mutations that were not selected in any of the threefold of the cross-validation were considered non-predictive for resistance.

### Statistical Analyses

Significant over-representation of premature stop codons in resistant isolates was assessed with Pearson’s Chi-squared test on a 2 × 2 matrix using the statistical software R (Version 3.4.0). PointFinder was compared with a similar predictor called PhyResSE. We assessed if PhyResSE performed significantly better than PointFinder using bootstrapping.

## Results

**FIGURE 1:**
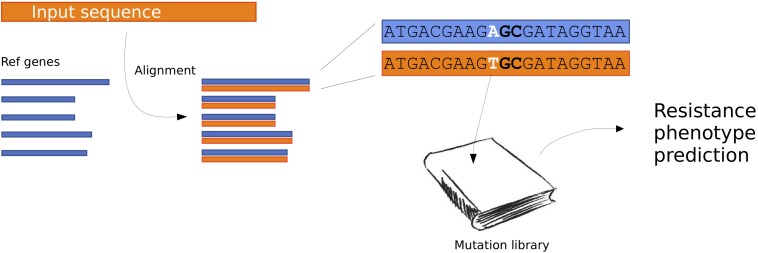
Flow chart describing the PointFinder workflow. The input sequences are aligned to a database of reference genes. The genetic differences observed in the alignments are compared to a mutation library, with annotated phenotypes. Based on this a resistance phenotype prediction is made.

We created an updated method for predicting antimicrobial resistance from the genomic sequence. An overview of the method can be seen in Figure 1.

### Evaluating and Optimizing the Mutation Library

We calculated the sensitivity, specificity and MCC for predicting drug resistance using PointFinder compared to phenotypic DST results (Table 2). The resistance prediction was based on mutations from the mutation library detected in the 3,528 *M. tuberculosis* isolates from the ReSeq data set. The best prediction performances were obtained for the first-line drugs Rifampicin, Isoniazid and MDR (MCC of 0.85, 0.82, and 0.86, respectively). PointFinder’s prediction performance varied dependently on the drug with MCCs ranging from 0.386 to 0.848. Especially, the prediction of resistance to Ethambutol, Pyrazinamide, Amikacin, and Ethionamide was less successful, which indicated that the mutation library was not fully developed.

**TABLE 2:** PointFinder predicted resistance compared with phenotypic drug susceptibility testing on the ReSeq data set.

**Drug**	**Res**	**Sus**	**Spec.**	**Sens.**	**MCC**
RMP	771	2710	0.965	0.887	0.848
INH	1093	2420	0.929	0.903	0.819
STM	728	1239	0.874	0.798	0.670
EMB	466	3040	0.796	0.850	0.484
PZA	325	2993	0.935	0.575	0.475
KAN	76	617	0.989	0.776	0.814
FLQ	240	1175	0.956	0.679	0.664
AMK	107	866	0.785	0.766	0.386
ETH	49	175	0.943	0.469	0.481
CAP	116	1024	0.971	0.474	0.512

*Res, number of resistant isolates determined by drug susceptibility testing; Sus, number of susceptible isolates determined by drug susceptibility testing; Spec, specificity; Sens, sensitivity; MCC, Mathew Correlation Coefficient; RMP, Rifampicin; INH, Isoniazid; STM, Streptomycin; EMB, Ethambutol; PZA, Pyrazinamide; KAN, Kanamycin; FLQ, Fluoroquinolones; AMK, Amikacin; ETH, Ethionamide; CAP, Capreomycin.*

Table 3 shows the occurrence of PointFinder-detected premature stop codons in the resistance-associated genes found in resistant and susceptible isolates. Genes shown in bold in Table 3 were in the mutation library described with position-specific premature stop codons causing resistance. With the exception of the panD gene, these genes showed a significantly higher occurrence of premature stop codons among resistant strains. However, for many genes the analysis was only based on a few premature stop codons. Only for four genes, katG, pncA, ethA, and gidB premature stop codons occurred more than 10 times, and we used this as a threshold for a considerable frequency. Moreover, premature stop codons in these genes were significantly over-represented in strains resistant to the drug that the genes were associated with (see Table 1). For katG, pncA, and ethA the representative *p*-values were below 0.00001 and for gidB it was 0.006. PointFinder’s prediction performance given in Table 4 shows that considering premature stop codons in the four genes as resistance markers improved the MCC of the resistance prediction for drugs in question; Isoniazid, Streptomycin, Pyrazinamide, and Ethionamide. In the case of Streptomycin and Ethionamide, the performances were improved with a compromise of the specificity.

**TABLE 3:** Occurrence of resistance-associated genes with premature stop codons in resistant or susceptible strains in the ReSeq data set.

**Drug**	**Res**	**Sus**	**Gene**	**Prem. stop codons in res. strains**	**Prem. stop codons in sus. strains**	***p*-value**
RMP	771	2710	rpoC	3	1	0.011
			**rpoB**	2	0	0.008^∗^
			inhA	1	0	0.137
INH	1093	2420	kasA	3	0	0.01^∗^
			ahpC	1	2	0.934
			**katG**	13	0	< 1.0e−5^∗^
STM	728	1239	rpsL	1	0	0.192
			gidB	42	40	0.006^∗^
			embA	1	4	0.658
			embR	3	4	0.021
EMB	466	3040	embC	1	4	0.658
			embB	1	2	0.306
			ubiA	1	0	0.011
PZA	325	2993	**pncA**	372	7	< 1.0e−5^∗^
			**panD**		6	0.148
ETH	49	175	inhA	1	0	0.058
			**ethA**	21	24	< 1.0e−5^∗^
CAP	116	1024	**tlyA**	2	2	0.008^∗^

*Genes annotated with premature stop codons in the mutation library are shown in bold. It was tested whether genes with premature stop codons were equally distributed between the two phenotypic groups. This was tested using a χ^2^ test on a 2 × 2 matrix, and the *p*-value is given. ^∗^*p*-value below the significance level 0.01. Res, population of resistant strains; Sus, population of susceptible strains. RMP, Rifampicin; INH, Isoniazid; STM, Streptomycin; EMB, Ethambutol; PZA, Pyrazinamide; ETH, Ethionamide; CAP, Capreomycin.*

**TABLE 4:** PointFinder predicted resistance compared with phenotypic drug susceptibility testing on the ReSeq data set when considering premature stop codons in katG, pncA, ethA, and gidB as resistance markers.

**Drug**	**Res**	**Sus**	**Spec.**	**Sens.**	**MCC**
RMP	771	2710	0.965	0.887	0.848
INH	1093	2420	0.929	0.909	0.823
STM	728	1239	0.847	0.839	0.674
EMB	466	3040	0.796	0.850	0.484
PZA	325	2993	0.935	0.612	0.502
KAN	76	617	0.989	0.776	0.814
FLQ	240	1175	0.956	0.679	0.664
AMK	107	866	0.785	0.766	0.386
ETH	50	175	0.829	0.800	0.564
CAP	116	1024	0.971	0.474	0.512

*Green indicates an increase and Red a decrease in performance compared to the initial prediction in Table 2. Res, number of resistant strains determined by drug susceptibility testing; Sus, number of susceptible strains determined by drug susceptibility testing; Spec, specificity; Sens, sensitivity; MCC, Mathew Correlation Coefficient; RMP, Rifampicin; INH, Isoniazid; STM, Streptomycin; EMB, Ethambutol; PZA, Pyrazinamide; KAN, Kanamycin; FLQ, Fluoroquinolone; AMK, Amikacin; ETH, Ethionamide; CAP, Capreomycin.*

Besides a possible lack of predictive premature stop codons, the mutation library also seemed to include mutations that were not predictive for resistance. For example, a low specificity was observed in the resistance prediction of Ethambutol and Amikacin, due to many false positive predictions. This indicated that the mutation library contained mutations, which should be omitted.

To detect such non-predictive mutations, we used a forward feature selection approach where the selection of mutations was optimized based the MCC over threefold cross-validation. Mutations not selected in any of the threefold of the cross-validation were considered non-predictive for resistance and shown in bold in Table 5. For 7 out of the 10 drugs, we found one or more mutations that were deselected in every fold and these mutations were omitted from the mutation library. The occurrence of the deselected mutations in resistant and susceptible isolates are shown in Supplementary Table S3.

**TABLE 5:** Forward feature selection of resistance mutations on the ReSeq data set.

**Drug**	**CV-fold**	**Deselected mutations**	**MCC train**	**MCC test**
	1	**rpoB I491F, rpoB H445N, rpoB L430P,** rpoC L527V	0.878	0.860
RMP	2	**rpoB 149IF, rpoB H445N, rpoB L430P,** rpoC L527V	0.868	0.878
	3	**rpoB 149 IF, rpoB H445N, rpoB L430P**	0.869	0.874
	1	**kasA G269S, kasA G312S, inhA V78A,** fabGl promoter −8T > C	0.880	0.884
INH	2	**kasA G269S, kasA G312S, inhA V78A**	0.881	0.882
	3	**kasA G269S, kasA G312S, inhA V78A**	0.883	0.877
	1	**rrs 1401A > G, rrs 492C > T**	0.743	0.744
STM	2	**rrs 1401A > G, rrs 492C > T**, gidB prem. stop codon	0.733	0.762
	3	**rrs 1401 A > G, rrs 492C > T**, gidB prem. stop codon	0.757	0.715
	1	**embB E378A, embC T270I, embB T1082A,**	0.648	0.590
		embA promoter −12C > T, embA promoter −16C > T		
EMB	2	**embB E378A, embC T270I, embB T1082A,** embB G406D, embB D1024N, embB N296H	0.643	0.616
	3	**embB E378A, embC T270I, embB T1082A,** embB G406D, embB N296H	0.623	0.660
	1	**pncA I6L,** pncA A146T, pncA W68G	0.589	0.625
PZA	2	**pncA I6L,** pncA A146T	0.590	0.636
	3	**pncA I6L**	0.638	0.537
	1		0.789	0.864
KAN	2		0.829	0.785
	3		0.826	0.795
	1	**gyrA T80A**	0.699	0.691
FLQ	2	**gyrA T80A**	0.686	0.713
	3	**gyrA T80A**	0.702	0.687
	1	**rrs 517C > T, rrs 514A > C**	0.702	0.744
AMK	2	**rrs 517C > T, rrs 514A > C**	0.722	0.705
	3	**rrs 517C > T, rrs 514A > C**	0.725	0.699
	1		0.652	0.393
ETH	2	ethA prem. stop codon	0.514	0.407
	3		0.543	0.592
	1		0.511	0.514
CAP	2		0.511	0.514
	3		0.514	0.509

*For all 10 drugs forward feature selection was performed over a threefold cross-validation to assess if the resistance performance could improve by omitting any mutations. CV-fold indicates for each fold of the cross-validation which mutations were deselected. Mutations shown in bold were deselected in every fold of the cross-validation and further considered non-predictive for resistance. MCC train, Mathew’s Correlation Coefficient obtained on the training data subset; MCC test, Mathew Correlation Coefficient obtained on the test data subset; RMP, Rifampicin; INH, Isoniazid; STM, Streptomycin; EMB, Ethambutol; PZA, Pyrazinamide; KAN, Kanamycin; FLQ, Fluoroquinolone; AMK, Amikacin; ETH, Ethionamide; CAP, Capreomycin.*

Table 6 shows the prediction performance when excluding the mutations from the mutation library. Omitting the non-predictive mutations from the mutation library did compromises the sensitivity, yet since the forward feature selection was trained based on the MCCs, the MCC performance was improved for all seven drugs.

**TABLE 6:** PointFinder predicted resistance compared with phenotypic drug susceptibility testing on the ReSeq data set after including premature stop codons and exclusion of non-predictive mutations.

**Drug**	**Res**	**Sus**	**Spec.**	**Sens.**	**MCC**
RMP	771	2710	0.978	0.878	0.871
INH	1093	2420	0.974	0.895	0.881
STM	728	1239	0.907	0.835	0.743
EMB	466	3040	0.898	0.848	0.631
PZA	325	2993	0.974	0.575	0.604
KAN	76	617	0.992	0.776	0.814
FLQ	240	1175	0.968	0.679	0.695
AMK	107	866	0.992	0.607	0.716
ETH	50	175	0.829	0.800	0.564
CAP	116	1024	0.971	0.474	0.512

*Green indicates an increase and red a decrease in performance compared to the prediction in Table 2. Res, number of resistant strains determined by drug susceptibility testing; Sus, number of susceptible strains determined by drug susceptibility testing; Spec, specificity; Sens, sensitivity; MCC, Mathew Correlation Coefficient; RMP, Rifampicin; INH, Isoniazid; STM, Streptomycin; EMB, Ethambutol; PZA, Pyrazinamide; KAN, Kanamycin; FLQ Fluoroquinolone. AMK, Amikacin; ETH, Ethionamide; CAP, Capreomycin.*

### Validating the Mutation Library Optimization

To validate the effects of including premature stop codons and excluding non-predictive mutations from the mutation library, we performed resistance predictions on a validation data set. This data set consisted of 2,480 isolates, and was independent of the ReSeq data set.

First, we examined occurrence of genes with premature stop codons in resistant and susceptible strains (Table 7). Like in the ReSeq data set premature stop codons occurred with a considerably frequency in the genes katG, pncA, ethA, and gibB. However, here only the katG and pncA premature stop codons were significantly over-represented in the resistant strains. gidB was close to the significant level of 0.01 (*p*-value: 0.015) whereas ethA premature stop codons seemed to be equally distributed between resistant and susceptible strains (*p*-value: 0.642).

**TABLE 7:** Occurrence of resistance-associated genes with premature stop codons found in resistant or susceptible strains in the validation data set.

**Drug**	**Res**	**Sus**	**Loci**	**Prem. stop codons in resistant strains**	**Prem. stop codons in susceptible strains**	***p*-value**
RMP	596	1814	rpoC	1	1	0.407
INH	768	1641	ahpC	0	1	0.566
			katG	8	2	0.001^∗^
STM	379	687	gidB	28	27	0.015
PZA	248	420	pncA	25	0	<1.0e−5^∗^
ETH	186	245	ethR	0	1	0.383
			ethA	24	28	0.642
CAP	191	261	tlyA	0	1	0.392

*It was tested whether genes with premature stop codons were equally distributed between the two phenotypic groups. This was tested using a χ^2^ test on a 2 × 2 matrix, and the *p*-value is given. ^∗^*p*-value below the significance level of 0.01. Res, number of resistant strains determined by drug susceptibility testing; Sus, number of susceptible strains determined by drug susceptibility testing; RMP, Rifampicin; INH, Isoniazid; STM, Streptomycin; PZA, Pyrazinamide; ETH, Ethionamide; CAP, Capreomycin.*

Additionally, we looked at the occurrence of the mutations that were considered non-predictive in the forward feature selection analysis. Data is shown in Supplementary Table S3. Most of the mutations that were found to be non-predictive for resistance in the ReSeq data set were confirmed to be widely present in susceptible strains in the validation data set. The mutations, rpoB I491F, inhA V78A, pncA I6L, gyrA T80A, and rrs 517C > T, were present in none or in very few samples in the validation data set, and therefore, the positive effect of removing these mutations could not be validated.

Table 8 shows prediction performances on the validation data set using three different mutation libraries; first, the initial mutation library, secondly, the mutation library where premature stop codons in katG, pncA, ethA, and gidB were included as resistance markers, and thirdly, the mutation library containing both the four premature stop codon markers and excluding the non-predictive mutations. Table 8 shows an overall improved prediction performance when including the premature stop codons as resistance markers and when excluding the non-predictive mutations.

**TABLE 8:** Validating the effect of including premature stop codons and excluding non-predictive mutations from the mutation library.

**Drug**	**Res**	**Sus**	**Initial mutations**			**Including prem. stop codons**			**Excluding non-predictive muts**		
			**Spec.**	**Sens.**	**MCC**	**Spec.**	**Sens.**	**MCC**	**Spec.**	**Sens.**	**MCC**
RMP	596	1814	0.978	0.896	0.885	0.978	0.896	0.885	0.986	0.886	0.895
INH	768	1641	0.946	0.879	0.826	0.945	0.880	0.826	0.969	0.870	0.854
STM	379	687	0.85	0.744	0.592	0.817	0.805	0.606	0.902	0.778	0.688
EMB	304	880	0.739	0.914	0.576	0.739	0.914	0.576	0.825	0.908	0.666
PZA	239	420	0.971	0.799	0.802	0.971	0.808	0.809	0.971	0.808	0.809
KAN	211	176	0.920	0.896	0.814	0.920	0.896	0.814	0.920	0.896	0.814
FLQ	271	296	0.905	0.878	0.784	0.905	0.878	0.784	0.909	0.878	0.788
AMK	213	278	0.953	0.850	0.814	0.953	0.850	0.814	0.964	0.826	0.807
ETH	184	245	0.624	0.853	0.479	0.539	0.919	0.479	0.539	0.919	0.479
CAP	191	261	0.877	0.864	0.738	0.877	0.864	0.738	0.877	0.864	0.738

*PointFinder-predicted resistance was compared to drug susceptibility test results on the validation data set. The quality of the prediction was evaluated with three different mutation libraries; the initial mutation library (initial mutations). The mutation library including premature stop codons in katG, pncA, ethA, and gidB (including prem. stop codons), and the mutation library both including those premature stop codons and excluding mutations considered non-predictive for resistance (excluding non-predictive muts). Green indicates an increase and red a decrease in the performance measurement compared to same measurement from the mutation library to the left. Res, number of resistant strains determined by drug susceptibility testing; Sus, number of susceptible strains determined by drug susceptibility testing; Spec, specificity; Sens, sensitivity; MCC, Mathew Correlation Coefficient; RMP, Rifampicin; INH, Isoniazid; STM, Streptomycin; EMB, Ethambutol; PZA, Pyrazinamide; KAN, Kanamycin; FLQ Fluoroquinolone; AMK, Amikacin; ETH, Ethionamide; CAP, Capreomycin.*

### Comparing PointFinder With Similar Tools

A scientific report from 2017 by Schleusener et al. PhyResSE generally showed the best performance, therefor we used the same data set to compare PointFinder to PhyResSE. We reran the data set through PhyResSE, to make a direct comparison. Table 9 show the prediction performance of PointFinder and PhyResSE based on WGS data and DST results from the 91 isolates. The mutation library used for PointFinder included premature stop codons in katG, pncA, ethA, and gidB and did not contain the non-predictive mutations. For Isoniazid, Streptomycin, and Ethambutol PhyResSE showed better performances. In the case of Streptomycin PheResSE performed significantly better than PointFinder which had a few false negative prediction, see Supplementary Table S4. For the drugs Rifampicin and Pyrazinamide PointFinder showed the best performance.

**TABLE 9:** Comparing PointFinder and PhyResSE prediction performance.

**Drug**	**Res**	**Sus**	**PointFinder**			**PhyResSE**			***p*-value**
			**Spec.**	**Sens.**	**MCC**	**Spec.**	**Sens.**	**MCC**	
RMP	14	77	0.961	1.000	0.890	0.935	1.000	0.830	0.751
INH	29	62	0.952	0.897	0.848	0.968	0.931	0.899	0.262
STM	37	54	0.981	0.649	0.693	0.981	0.838	0.843	0.047^∗^
EMB	14	77	0.961	0.857	0.796	0.974	0.857	0.831	0.395
PZA	8!	83!	0.964	0.750	0.677	0.964	0.625!	0.589	0.666

*! Indicates that data varied from the previously published results, since the number of resistance and susceptible samples found in the published Supplementary Data did not correlate with amount assessed after rerunning the data through PheResSE. Res, number of resistant strains determined by drug susceptibility testing; Sus, number of susceptible strains determined by drug susceptibility testing; Spec, specificity; Sens, sensitivity; MCC, Mathew Correlation Coefficient; RMP, Rifampicin; INH, Isoniazid; STM, Streptomycin; EMB, Ethambutol; PZA, Pyrazinamide. It was tested weather PhyResSE was performing significantly better using bootstrapping, and the *p*-value is given, and the significance level was set at 0.05 indicated with ^∗^.*

## Discussion

In this study, we presented an improved version of PointFinder where the detection of insertion and deletion together with frameshift mutations were handled properly. As an effect of the improvements we were able to enhance PointFinder’s resistance prediction *in M. tuberculosis* by including premature stop codons as resistance markers. Additionally, we optimized the obtained *M. tuberculosis* mutation library by excluding mutations that through forward feature selection were considered non-predictive for resistance.

A scientific report from 2017 by Schleusener et al. compared five *M. tuberculosis* resistance prediction tools based on a data set of 91 isolates. These five tools were, CASTB (Iwai et al., 2015), PhyResSE (Feuerriegel et al., 2015), TBProfiler (Coll et al., 2015), KvarQ (Steiner et al., 2014), and Mykrobe Predictor TB (Bradley et al., 2015). To our knowledge it has not been studied thoroughly how the occurrence of premature stop codons in resistance-associated genes affect the resistance phenotype. The mutation library lists premature stop codons predictive for resistance, yet these premature stop codons are only considered as predictive markers if found at the specific position listed. However, the outcome of a premature stop codon – gene truncation – is, in most cases, independent of the position in the gene. The first version of PointFinder described in Zankari et al. (2017) did not consider insertions and deletions, and as a consequence of this, frameshift mutations and premature stop codons was not correctly detected. With this new version of PointFinder, efforts were put into detecting reading frame disruptions and premature stop codons caused by insertions and deletions, and the improved PointFinder version was used to assess the impact of premature stop codons on resistance emergence.

Among all genes annotated with predictive premature stop codons in the mutation library we found a significantly higher occurrence of premature stop codons among resistant strains in the ReSeq data set, with the exception of the panD gene (Table 3). A study from 2014, showed a *M. tuberculosis* panD deleted mutant still susceptible to Pyrazinamide (Dillon et al., 2014). The study postulated that panD is not a target for Pyrazinamide resistance, and our results support this hypothesis by indicating that loss of function of panD is not associated with Pyrazinamide resistance.

Our results suggest that katG and pncA premature stop codons are predictive for resistance, whereas the role of ethA and gidB premature stop codons was less clear. Isoniazid, Pyrazinamide, and Ethionamide are pro-drugs, and the proteins encoded by katG, pncA, and ethA are enzymes catalyzing the activation of these drugs, respectively (Zhang et al., 1992; Almeida Da Silva and Palomino, 2011). If the enzymatic activity is lost (e.g., by the occurrence of a premature stop codon), the drug cannot be converted to its active form, which can explain the emergence of drug resistance.

Surprisingly, premature stop codons in ethA also occurred with a high frequency in susceptible strains, and in the validation data set premature stop codons in ethA were not over-represented in resistant strains (Table 7). Since, ethA encodes the Ethionamide activating enzyme, we speculate whether this is not the only enzyme able to activate Ethionamide, or if Ethionamide also has antimicrobial effects as a pro-drug, or maybe premature stop codons can be neglected and not cause complete depletion of the ethA-encoded enzyme. Another explanation for the inconclusive effect of ethA premature stop codons, might be that the use of Ethionamide constitutes a selective pressure that favors premature stop codon in ethA leading to low-levels resistance close to the clinical breakpoint used in DST protocols.

Premature stop codons in gidB were slightly over-represented among the resistant strains both in the ReSeq (*p*-value = 0.006) and the validation data set (*p*-value = 0.015), yet, premature stop codons in gidB were also observed in many susceptible isolates (see Tables 3, 7). Like for ethA, this might reflect that depletion of the gidB-encoded protein causes resistance levels close to the clinical breakpoint. In fact, a functional study showed that knocking out gidB leads to low-level Streptomycin resistance (Wong et al., 2011). We observed an increased MCC when treating the mutation as a resistance marker, thereby, our study also indicates that loss of function of the gidB-encoded protein is associated with Streptomycin resistance.

The forward feature selection analysis implied that several mutations included in the obtained mutation library were misclassified as resistance markers, and the positive effects of removing these mutations were also seen in the MCC on the validation data set (Table 8), with the exception of predicting Amikacin resistance. The two mutation rrs 514A > C and rrs 517C > T that were removed in this case, were however also found in other studies to play no role in resistance to Amikacin (Maus et al., 2005; Jugheli et al., 2009).

Further investigation showed that the misclassification of many of the deselected mutations was also reported in other studies, for example for kasA G269S and kasA G312S (Sun et al., 2007), rrs 492C > T (Victor et al., 2001; Villellas et al., 2013), rrs 1401A > G (for Streptomycin resistance) (Via et al., 2010), gyrA T80A (Pantel et al., 2016), and embB E378A and embC T270I (Goude et al., 2009; Campbell et al., 2011; Koser et al., 2011). In the forward feature selection analysis, we chose to only include mutations that were observed 10 times or more, however, with more isolates or a lower threshold for including mutations, we might discover even more misclassified mutations. On the other hand, the mutation rpoB L430P, rpoB H445N, and rpoB I491F were considered non-predictive for resistance to Rifampicin based on the forward feature selection. However, studies have shown that DST performed on liquored-based mediums fails to detect resistance in strains with rpoB I491F and other rpoB mutations that were clinically associated with treatment failure (Rigouts et al., 2013; André et al., 2017). Thus, with forward feature selection we risk removing mutations that truly causes resistance but appears not to, due to erroneous DST results. This underlines a problem regarding using DST results as the standard for determining resistance. A well-established mutation library is important to avoid incorrect mutation interpretations.

When comparing PointFinder to PhyResSE we did see differences in variant interpretation. This was notable in the gidB gene associated with Streptomycin resistance. PointFinder only interpreted resistance based on premature stop codons in gidB, whereas PhyResSE included several gidB mutations in the interpretation, e.g., gidB A200E, V88A, and A138V (see Supplementary Table S4), and with the interpretation of these mutations as resistance markers PhyResSE showed a significantly better Streptomycin resistance prediction. A GWA study from 2018 did detect the same gidB mutations among 6,465 strains, but in this study this gidB mutations were either observed in less than 10 samples or not identified as being significantly associated with resistance (Coll et al., 2018). Based on this, we did not choose to include these gidB mutation in our mutation library. We have here evaluated the effect of genetic alterations on resistance. A limitation of this is that it is overlooked if mutations have an effect of for example fitness. Future studies may seek to clarify such correlations if large scale datasets with genomes and fitness estimations become available.

The predicting performance of PointFinder is comparable to other *M. tuberculosis* resistance prediction tools, like PhyResSE, and PointFinder has the advantage of being build into a larger platform for resistance prediction, that is not limited to a single species. Additionally, PointFinder is available on bitbucket.org/genomicepi-demiology/pointfinder, where all changes in the script are tracked. The databases are also available on bitbucket which gives the needed transparency. This creates a good foundation for future maintenance and improvements of the variant interpretation methods and the mutation library.

## Conclusion

We have developed improved version of PointFinder with better detection of insertions and deletions as well as the possible associated frameshifts. We find that the accuracy of PointFinder’s resistance prediction in *M. tuberculosis* is improved as a result. We also optimized the *M. tuberculosis* mutation library by excluding mutations that through forward feature selection were found to be non-predictive for resistance. We think that these methods may also be applied to increase the antibiotic resistance prediction in other species. The method is flexible and can be updated if new genetic markers for resistance is identified. The method is freely available on the web as well as a stand alone version.

## Data Availability Statement

The datasets analyzed in this study was obtained from platform.reseqtb.org and as Supplementary Data from Coll et al. (2018) and Schleusener et al. (2017). All accession numbers and phenotype data are also given as Supplementary Data (Supplementary Tables S1, S2, S4).

## Author Contributions

CJ implemented changes in the improved version of PointFinder, performed all analyses, and wrote the manuscript with inputs from all authors. PC provided help with statistical calculations and did proofreading. OL supervised the project and, together with FA, were in charge of overall direction and planning.

## Conflict of Interest

The authors declare that the research was conducted in the absence of any commercial or financial relationships that could be construed as a potential conflict of interest.

## Supplementary Material

TABLE S1 ReSeq data set.

TABLE S2 Validation data set.

TABLE S3 Occurrence of non-predictive mutation in the ReSeq and validation data set.

TABLE S4 Comparison of PointFinder and PhyResSE.
