# Supplementary material for: Improved Resistance Prediction in Mycobacterium tuberculosis by Better Handling of Insertions and Deletions, Premature Stop Codons, and Filtering of Non-informative Sites
Source: Front Microbiol. 2019 Oct 31;10:2464. doi: 10.3389/fmicb.2019.02464 (PMC6834686; doi:10.3389/fmicb.2019.02464)
Supplement: TABLE S1 — ReSeq data set. [file Data_Sheet_1.ZIP › supplementary/S3.pdf]

Occurrence of non-predictive mutation in the ReSeq and validation data set

Drug	Mutation	Occur in sus strains	Occur in res strains	TN gain	FN gain
RMP	rpoB I491F	18	4	18	4
	rpoB H445N	6	7	6	1
	rpoB L430P	13	9	13	1
				37	7
INH	kasA G269S	77	49	77	14
	kasA G312S	22	9	21	0
	inhA V78A	13	4	13	1
				111	15
STM	rrs 1401A>G	17	68	9	2
	rrs 492C>T	66	1	66	1
				75	3
EMB	embB E378A	308	12	0	0
	embC T270I	308	12	0	0
	embB T1082A	10	1	10	1
				308	1
PZA	pncA I6L	120	12	119	12
FLQ	gyrA T80A	15	0	14	0
AMK	rrs 517C>T	163	15	163	15
	rrs 514A>C	16	12	16	2
				179	17

Table S3.1: Non-predictive mutation evaluated by forward selection of mutation on the ReSeq data set. *Forward selection of mutations predictive for resistance was performed on the ReSeq data set. Mutations that were not selected in any of the 3 folds of cross validation are listed. For each mutation the number of times it occurred in susceptible (Occur in sus strains) and resistant strains (Occur in res strains) are given. The gain of true negatives (TN gain) and false negatives (FN gain) when omitting each mutations are shown. If applicable, the total TN gain and FN gain are shown after of discarding all the mutations as predictive markers of resistance to the drug. RMP, Rifampicin; INH, Isoniazid; STM, Streptomycin; EMB, Ethambutol; PZA, Pyrazinamide; FLQ Flouroquinolones; AMK, Amikacin.*

Drug	Mutation	Occur in sus strains	Occur in res strains	TN gain	FN gain
RMP	rpoB I491F	0	2	0	2
	rpoB H445N	13	2	13	1
	rpoB L430P	2	5	2	3
				15	6
INH	kasA G269S	49	50	33	8
	kasA G312S	6	2	6	0
	inhA V78A	0	0	0	0
				39	8
STM	rrs 1401A>G	47	109	32	7
	rrs 492C>T	28	5	25	2
				59	10
EMB	embB E378A	81	9	0	0
	embC T270I	81	9	0	0
	embB T1082A	3	1	1	1
				76	2
PZA	pncA I6L	0	0	0	0
FLQ	gyrA T80A	1	0	1	0
AMK	rrs 517C>T	0	1	0	1
	rrs 514A>C	5	81	3	4
				3	5

Table S3.2: Occurrence of non-predictive mutations in the validation data set. *For each mutation the number of it occurrence in susceptible (Occur in sus strains) and resistant strains (Occur in res strains) are given. The gain of true negatives (TN gain) and false negatives (FN gain) when omitting the mutations are shown. If applicable, the total TN gain and FN gain are shown after of discarding all the mutations as predictive markers of resistance to the drug. RMP, Rifampicin; INH, Isoniazid; STM, Streptomycin; EMB, Ethambutol; PZA, Pyrazinamide; FLQ Flouroquinolones; AMK, Amikacin.*
